# Case report of a brain abscess due to *Nannizziopsis obscura* species complex in a renal transplanted patient

**DOI:** 10.1016/j.mmcr.2026.100821

**Published:** 2026-09-04

**Authors:** Othman Mounir Alaoui, Astrid De Liège, Frédéric Mechai, Dea Garcia-Hermoso, Fanny Lanternier, Bertrand Degos

**Affiliations:** a Service de Neurologie, APHP, Hôpital Avicenne, Hôpitaux Universitaires de Paris - Seine Saint Denis, Sorbonne Paris Nord, Bobigny, France; b Service de Maladies Infectieuses et Tropicales, APHP, Hôpital Avicenne, Hôpitaux Universitaires de Paris - Seine Saint Denis, Sorbonne Paris Nord, Bobigny, France; c Institut Pasteur, Université Paris Cité, National Reference Center for Invasive Mycoses and Antifungals, Translational Mycology Research Group, Mycology Department, Paris, France; d Center for Interdisciplinary Research in Biology (CIRB), Collège de France, CNRS, INSERM, Université PSL, Paris, France

**Keywords:** Nannizziopsis, Brain abscess, Immunosupression, Fungal infection

## Abstract

We here describe the case of a kidney transplant patient from Mali who presented with multiple complex seizures followed by a facial paresis revealing a brain abscess due to *Nannizziopsis obscura* species complex. This fungal species is usually implicated in reptile skin infections and rarely disseminated infections. Very rarely, it can infect human beings suffering from immunodepression, causing various kinds of complications ranging from soft tissue abscesses to solid organ abscesses. This case highlights the potential wide range of pathogens involved and underlines the need for greater vigilance in the diagnosis and management of atypical infections in transplant settings.

## Abbreviation:

CMV: cytomegalovirus
CSF: cerebrospinal fluid
EBV: Ebstein-Barr virus
HBV: Hepatitis B virus
HIV: human immunodeficiency viruses
HHV: Human Herpesvirus
HSV: Herpes simplex virus
MRI: magnetic resonance imagery
*N*: *Nanniziopsis*
PCR: polymerase chain reaction
Spp: *species plurimae*

## 1 Introduction

Brain abscesses are a rare but serious complication of solid organ transplantation due to the drug-induced immunosuppression required to prevent transplant rejection. It is estimated to occur in less than 1% of transplant patients, with high mortality rates [1], [2], [3].

Identification of the causative pathogen is crucial to provide the right treatment and improve outcomes. However, this process can be quite difficult because the clinical symptoms and paraclinical examinations may not be very specific [4], [5].

*Nannizziopsis* spp. is an emerging infectious pathogen affecting reptiles and amphibians with a tropism for keratin, causing skin infections that sometimes extend to several organs [6]. It is exceptionally associated with human infections and only a few cases have been reported (see Table 1). Here, we describe the case of a brain abscess caused by *N. obscura* in a patient who had undergone a kidney transplant.

**Table 1:** Characteristics of all reported cases of *Nannizziopsis* infections.

Date	Gender	Age	Context and risk factors	Origin	Affected organ	Diagnosis	Isolated species	Treatment	Follow-up
1982 [7], [8]	Male	24	Unknown	Trips to Africa	Abscess on right ankle + osteomyelitis	Abscess biopsy	*N. obscura*	Amphotericin B	Cured at M4, persistent lytic area on X-ray at Y2
1994 [8]	Male	N/A	HIV+	N/A	Mass in right thigh, lungs	Muscle biopsy	*N. hominis*	Itraconazole	Death after 8 months probably from HIV complications
2000 [8]	Male	32	Immunocompetent	Trips to Nigeria	Lymph nodes, heart, lungs, spleen, kidneys	Lymph nodes biopsy	*N. hominis*	Itraconazole for 2 years	Unknown
2004 [9]	Male	49	HIV+ 0 CD4	Mali	Liver abscess	Needle aspiration of the liver	*N. obscura*	Amphotericin B + Metronidazole at least 15 days	No follow-up
2005 [10]	Male	38	HIV+; 102 CD4	Nigeria	Brain abscess	Needle aspiration of brain lesion	*Chrysosporium* anamorph of *N. vriesii*	Voriconazole + aspiration	At M4: in good health with no neurological deficit, partial regression of brain lesions
2005 [11], [12]	Male	40	HIV+; 13 CD4	Unknown	Lungs	Bronchial washing	*Chrysosporium* anamorph of *N. vriesii*	No treatment	No follow-up
2009 [9]	Male	50	Heart transplantation (2009) Prednisone 15mg + Ciclosporin	Mali	Multiple organ damage	Blood culture	*N. obscura*	No treatment	Death before treatment
2015 [12]	Female	63	T-cell prolymphotic leukemia (2014) treated with Alemtuzumab then Bendamustine then Idelalisib	Several trips to Senegal	Brain abscess	Blood culture, CSF, ascites fluid	*N. obscura*	Death before antifungal treatment	Death before antifungal treatment
2015 [13]	Male	34	Kidney transplantation Tacrolimus, MMF, Prednisone 5mg	Gambia	Chronic subcarinal lymphadenopathy (2 months), pulmonary nodules, soft tissue collection in T6-T8 then collection of thoracic musculature	Surgical drainage of the thoracic muscle collection + lymph node biopsy	*N. obscura*	Surgical drainage + Posaconazole for 10 months	Improvement of thoracic collection and skin lesions at M3, no evidence of recurrence at M17
2017 [9]	Female	58	Kidney transplantation (2016) Tacrolimus, MMF, Prednisone 5mg	Mali	Lung nodule + nodular skin lesions on both legs	Skin and lung biopsy	*N. obscura*	Voriconazole then Posaconazole for 8 months + reduction of Tacrolimus and switch MMF for Azathioprine	Residual hypermetabolic lesion at M6, end of treatment at M8, no relapse at Y1
2017 [9]	Male	62	Kidney transplantation (2009) Tacrolimus, MMF, Prednisone 5mg	Guinea	Suppurated lesions on the right fibula	Direct examination of infected lesions	*N. obscura*	Voriconazole then posaconazole for 2 years	Residual hypermetabolism of the right ankle at M6, no hypermetabolism at Y2
2017 [12]	Female	52	HIV+; 3 CD4	Mali	Brain abscess + nodular lesion of the right lung	Cerebral biopsy, CSF	Closely related to *N. obscura*	Fluconazole + Pyrimethamine-Sulfadiazine then Amphotericin then Voriconazole	M1: improvement of general status, regression of lung lesions and cerebral abscess
2018 [9]	Female	69	Advanced mantle cell lymphoma treated in 2017 T-cell lymphoma discovered during hospitalization	Guinea	Lung lesions + lymphadenopathy + nodular hyperchromic skin lesions	Skin biopsies	*N. obscura*	Voriconazole + Amphotericin B then died	Death despite intensive care and antifungal treatment
2018 [9]	Female	27	No notable medical history	Guinea	Mediastinal mass	Surgical resection	*N. obscura*	Posaconazole then Voriconazole duration unknown	Reduction in mass size at M7
2018 [9]	Male	38	Kidney transplantation (2015) Tacrolimus, MMF, Prednisone 5mg	Mali	Brain abscess	Cerebral biopsy	*N. obscura*	Amphotericin B + Fluconazole then Amphotericin B + voriconazole	Still alive at M12
2019 [9]	Male	79	Kidney transplantation (2014) Tacrolimus, MMF, Prednisone 5mg	Mali	Ulcerative lesion of the fifth right finger + pulmonary nodule	Skin biopsy	*N. obscura*	Itraconazole	Improvement at M4
2019 [9]	Male	65	Kidney transplantation (2018) Tacrolimus, MMF, Prednisone 5mg	Mali	Left clavicle abscess	Culture of pus from spontaneous fistula	*N. obscura*	Voriconazole	Unknown
2019 [14]	Male	56	Kidney transplantation (2013) Tacrolimus, MMF, Prednisone 5mg	Senegal	Ankle arthritis	Debridement of skin and subcutaneous tissue, histological examination + culture of pus	*N. obscura*	Surgery + Amphotericin B for 5 days then Voriconazole (ultra-rapid metabolizer profile) then Posaconazole for 1 year	No recurrence + partially functional right lower limb at Y1
2020 [15]	Female	13	STAT1 mutation	Nigeria	Brain abscess + osteomyelitis + pneumonia	Bone biopsy + cerebral biopsy	*Nanniziopsis* spp.	Ventricular drain + Amphotericin B then Voriconazole	Almost complete resolution of brain lesions at M4, removal of ventricular drain at M11
2021 [16]	Male	35	HIV+; 4 CD4	Togo	Brain abscess + cavitary lesion of the right upper lung	Bronchial washing	*Nanniziopsis* spp.	Voriconazole 12 weeks	Reduction in lesion size at M3, asymptomatic at M18
2021 [17]	Female	45	Kidney transplantation Prednisone 5 mg, Tacrolimus 9 mg - 8,5 mg, MMF 500 mg - 250 mg	Nigeria	Breast abscess	Needle biopsy	*N. obscura*	Drainage + Anidulafungin 100 mg IV 14 days + reducing the Tacrolimus dose to reach a target level of 4–5 ng/mL switch to Isavuconazole 600 mg for two days and a subsequent dose of 200 mg daily for a total of 14 more days	Unknown
2022	Male	54	Kidney transplantation Sirolimus 1.5mg Tacrolimus 7mg Prednisone 5mg	Mali	Brain abscess	Brain biopsy	*N. obscura*	Amphotericin B + Flucytosin then Voriconazole	Clinically asymptomatic and partial regression of the brain lesion at M8
2023 [18]	N/A	N/A	N/A	Venezuela	Tongue lesion	Biopsy ?	*N. arthrosporioides*	N/A	N/A

Abbreviations. CSF: cerebrospinal fluid; HIV: human immunodeficiency viruses; M: month; MMF: mofetil mycophenolate; N: Nannizziopsis; N/A: not available; Spp: species plurimae; Y: year.

## 2 Case presentation

In July 2022, a 54-year-old patient from Mali presented with multiple complex motor seizures followed by post-ictal right hemiplegia. The patient had a history of renal transplantation in 2017 after 4 years of dialysis due to nephroangiosclerosis. He suffered from multiple infectious complications due to his immunosuppression: pulmonary and lymphatic infection due to *Mycobacterium intracellulare*, CMV and HBV reactivation, and Kaposi disease with pulmonary, lymphatic and cutaneous localizations, all of which were considered as resolved before the onset of symptoms. In particular, to treat Kaposi disease, dermatologists decided to reduce immunosuppression by replacing mycophenolate mofetil with sirolimus in September 2019, but still in combination with tacrolimus and prednisone, resulting in a significant improvement seen after 3 months and persisting thereafter. On admission (Day 0), he was receiving immunosuppressive drugs (sirolimus 1.5mg, tacrolimus 7mg and prednisone 5mg, daily).

Initial management in the intensive care unit consisted of administering anti-epileptic drugs and undertaking diagnostic investigations. He was apyretic and had no new neurological symptoms on admission.

**Fig. 1:**
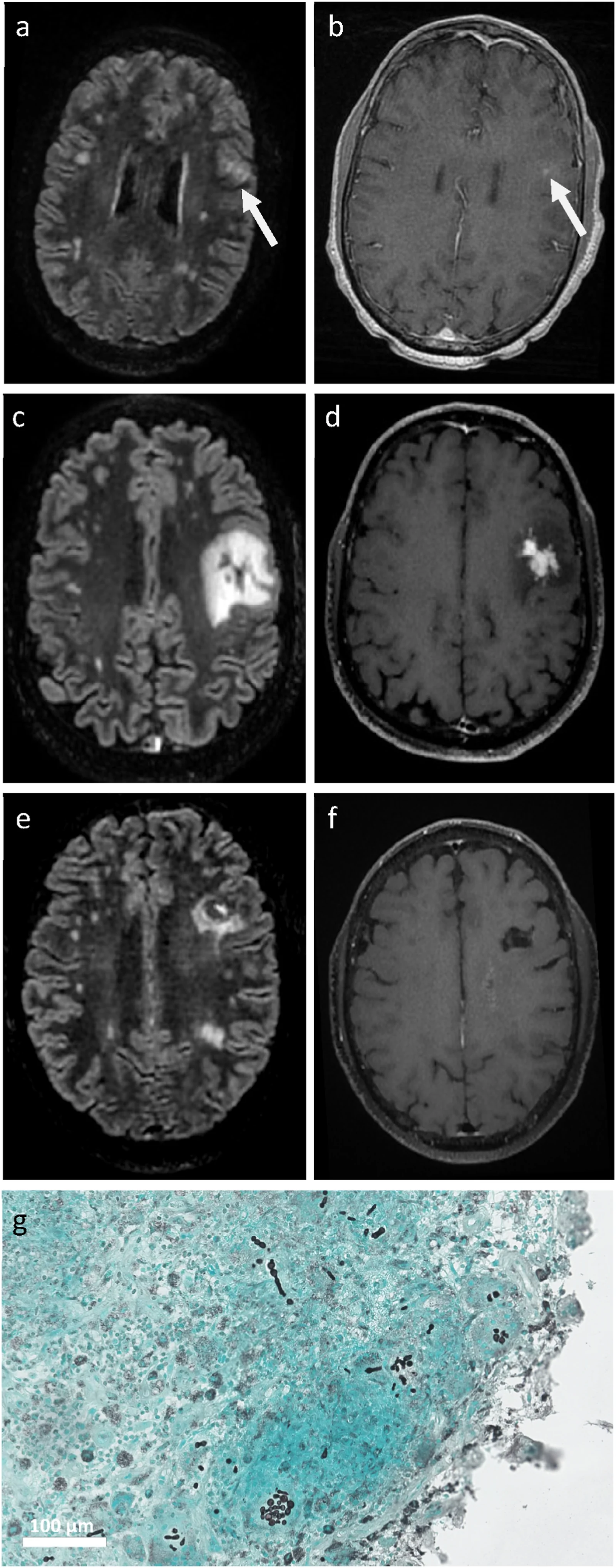
Brain Imagery and anatomopathological examination of the brain lesion a: Day 1, T2 FLAIR-weighted brain MRI showing a heterogenous left frontal cortico-subcortical hyperintensity (white arrow). b: Day 1, Gadolinium-injected T1-weighted brain MRI showing a contrast enhancement in the same region (white arrow). c: Day 18 (1 day after neurological worsening), T2 FLAIR-weighted brain MRI showing a large increase in lesion size with peri-lesional oedema. d: Day 18 (1 day after neurological worsening), Gadolinium-injected T1-weighted brain MRI showing increased contrast enhancement associated with a heterogeneous appearance. e: M8, T2-weighted FLAIR brain MRI showing a significant decrease in lesion size. f: M8, Gadolinium-injected T1-weighted brain MRI showing the disappearance of the contrast enhancement. g: Grocott–Gömöri's methenamine silver stain (black) of the brain biopsy demonstrating the presence of fungal elements.

Blood tests on admission showed no abnormalities, except for a known increase in creatinine level around 220 μmol/L (normal level < 115 μmol/L). A magnetic resonance imaging (MRI) displayed a discrete left frontal lesion with contrast enhancement on T1-weighted sequences, without diagnostic orientation (Fig. 1a and b).

Cerebrospinal fluid (CSF) analyses performed on day + 1 showed no pleocytosis nor hypoglycorrhachia. Polymerase chain reaction (PCR) for CMV, EBV, HSV1, HSV2, JC, HHV6, HHV8, enterovirus, *Toxoplasma gondii*, *Nocardia*, *Mycobacterium tuberculosis* was negative, as were bacterial cultures, *Aspergillus* (galactomannan) antigen and India ink stain for *Cryptococcus*.

On day + 5, the patient was transferred in our Neurology department at Avicenne University hospital for further examination. A thoraco-abdomino-pelvic computed tomography scan was performed on day + 6 and revealed a newly developed nodule in the lower lobe of the left lung, which had not previously been seen in January 2022, at the time of the last systematic evaluation after Kaposi disease. A bronchial fibroscopy performed on day + 7 revealed no signs of malignancy, and bacterial and mycobacterial cultures of bronchoalveolar lavage fluid were all negative. On day + 17, the patient presented a right central facial paralysis. MRI performed after this neurological worsening on day + 18 showed a significant increase in the size and contrast of the initial lesion (Fig. 1c and d), leading us to suspect an infectious disease as a first hypothesis. We especially suspected parasitic infections such as bilharzia, given the radiological characteristics of the lesion showing an ill-defined tumor-like enhancement and the presence of bladder calcifications. A second CSF analysis performed at day + 19 was normal. Control PCR for CMV, EBV, HSV1, HSV2, JC, HHV6, HHV8 and *Mycobacterium tuberculosis* were negative. Bacterial cultures were taken on day + 19 and were also negative. Immunophenotyping and interleukin assays revealed no evidence for neurolymphoma.

Stool, urine and blood analyses performed on day + 19 were also negative for parasitologic and mycological tests. Given the negativity of all blood and cerebral fluid tests, a neurosurgical biopsy was performed on day + 27 to identify the pathogen.

Histopathological examination of the brain biopsy showed numerous non-necrotizing epithelioid and multinucleated giant cell granulomas, associated with a mixed inflammatory infiltrate. Fungal elements were observed within the lesion (Fig. 1G). We therefore began treating the patient with amphotericin B 3mg/kg/24h and flucytosine 25mg/kg/12h on day + 28.

Fungal cultures resulted in the growth of yeasts and filamentous elements. However, morphological features were non-specific and did not allow species-level identification. MALDI-TOF mass spectrometry suggested identification within the genus *Nannizziopsis*. Species-level identification was achieved by molecular analysis as described in Garcia-Hermoso et al. [9]. Sequences of the internal transcribed spacer (ITS) region and of the actin gene were compared against curated fungal references databases using BLAST (https://blast.ncbi.nlm.nih.gov) allowing the identification of the species *obscura*.

In accordance with the literature, the patient was then treated with intravenous voriconazole at a loading dose of 6mg/kg/12h, followed by a maintenance dose of 4mg/kg/12h (220 mg/12h). Therapeutic drug monitoring revealed an insufficient initial serum level at 2.65 mg/L, prompting a dose adjustment to 4.5 mg/kg/12h (250 mg/12h). In vitro antifungal susceptibility testing of the isolate demonstrated minimum inhibitory concentrations of 0.5 mg/L for amphotericin B, 0.125 mg/L for itraconazole, 0.25 mg/L for voriconazole, 0.03 mg/L for posaconazole, 0.25 mg/L for isavuconazole, 1 mg/L for caspofungin and < 0.008 mg/L for micafungin. Subsequent monitoring showed a stable concentration of voriconazole within the target range between 3.5 and 5 mg/L. After a two weeks course of intravenous therapy, the patient was transitioned to oral voriconazole at a dose of 250 mg/12h prior to discharge, resulting in favorable clinical and radiological outcomes.

At the 8-month follow-up (M8), the patient was still under voriconazole 4mg/kg/12h. He was neurologically asymptomatic and brain MRI showed an improvement in the evolution of the lesions (Fig. 1e and f). Patient was then lost to follow-up.

## 3 Discussion

Brain abscesses are a rare but serious complication of solid organ transplantation. The most common clinical presentations are fever and altered mental status. Patients may also present with focal neurological symptoms or epileptic seizures, as in the case of our patient [4].

The epidemiology of brain abscesses in transplant patients seems to depend on the onset of infection in relation to the moment of transplantation: early-onset brain abscesses (less than 6 months after transplantation) are mainly due to fungal infections and late brain abscesses may be caused by other pathogens (*Nocardia*, *Toxoplasma gondii*) [1].

Fungal infections of the brain are difficult to diagnose because clinical symptoms and brain imaging are not very specific. CSF analysis can be very useful for assessing the presence of fungal antigens (galactomannan, β-D-Glucan) but definitive identification of the pathogen is often difficult. The search for other infected organs (lymph nodes, lungs, skin) with potential biopsy sites can help make the right diagnosis [5]. However, when first- and second-line investigations are negative, specialist discussions between neurologists, infectious disease specialists and neurosurgeons are needed to determine the indication for a brain biopsy. In our case, only a brain biopsy was able to identify the pathogen responsible for the brain abscess.

*Nannizziopsis* spp. are keratinophylic fungi first described from day geckos (*Phelsuma sp*.) in 1991. They have subsequently been described in several different reptile species. They mainly cause skin infections that can spread and lead to the failure of several organs [6].

Very rarely, these fungi can infect humans, leading to various localizations of infection, such as the lung, kidney, liver, spleen, brain, breast, lymph nodes, soft tissues, bones and articulations. To our knowledge, only 22 other cases of human *Nannizziopsis* infections have been described in the literature (Table 1), our patient being the second kidney transplant patient with a brain abscess due to *N. obscura*. The main risk factor appears to be immunosuppression, as only one reported case (5%) involved an immunocompetent patient at the onset of symptoms. All reported conditions (*i.e.* HIV infection, solid organ transplantation, T-cell lymphoma, STAT1 mutation) result predominantly in T-cells deficiencies rather than other kinds of immunodeficiency. In addition, almost all patients originate from, or have traveled to, West African countries. However, several years may elapse between the last trip in one of these countries and the onset of symptoms. Only one case has been reported in Venezuela, but no clinical data on other risk factors are available. As for our patient, his last trip to Mali was more than 4 years before his first symptoms. This raises the question whether patients carry a latent form of this fungus that reactivates upon immunosuppression. Diagnosis consistently relies on invasive sampling of the affected organs, including stereotactic brain biopsies, bone and skin biopsies, or deep aspiration cultures. *Nannizziopsis obscura* is the most frequently identified species, followed by *N. hominis* and *N. vriesii*. Macroscopically, *Nannizziopsis* isolates produce moderately fast-growing, yellowish, velvety to powdery colonies on PDA [7,9]. Under microscopy, they commonly exhibit sessile aleurioconidia with truncate bases. They typically have chains of cylindrical arthroconidia formed by schizolytic hyphal fragmentation; and short undulate lateral branches. However, these phenotypic traits overlap considerably across the genus, rendering DNA sequencing essential for definitive species identification. Antifungal management is heterogenous but centers predominantly on broad-spectrum azoles and amphotericin B formulations, frequently combined with surgical drainage or reduction of immunosuppressive therapy. Given the invasive nature and slow resolution of deep-seated and central nervous system lesions, prolonged antifungal courses are required, ranging from several months to over two years. When diagnosed early and with prolonged azole treatment, favorable clinical and radiological outcomes can be achieved. However, the prognosis remains guarded, with notable mortality especially in case of delayed antifungal therapy.

In conclusion, *Nannizziopsis* spp. Should be considered an emerging opportunistic pathogen in patients with marked cell-mediated immunosuppression with epidemiological links to West Africa, in whom early tissue-based diagnosis and extended triazole therapy are critical to improving clinical outcomes.

## CRediT authorship contribution statement

**Othman Mounir Alaoui:** Writing – original draft, Visualization, Investigation, Conceptualization. **Astrid De Liège:** Writing – review & editing, Investigation. **Frédéric Mechai:** Investigation. **Dea Garcia-Hermoso:** Writing – review & editing, Resources, Methodology. **Fanny Lanternier:** Resources, Methodology. **Bertrand Degos:** Writing – review & editing, Supervision, Conceptualization.

## Consent to publish:

The authors affirm that the patient provided informed consent for publication of this case and of Fig. 1.

## Data availability statement

The data are not available for public access because of patient privacy concerns but are available from the corresponding author BD on reasonable request.

## Consent

In accordance with French law, the patient was informed, via an information sheet, that his medical records might be used for research purposes, and all data that could potentially identify him were pseudonymised during the data extraction process in order to protect his confidentiality. Written informed consent was not required for this retrospective case study.

## Funding source

There are none.

## Conflict of interest

There are none.
